# Histological and Transcriptomic Analysis of Adult Japanese Medaka Sampled Onboard the International Space Station

**DOI:** 10.1371/journal.pone.0138799

**Published:** 2015-10-01

**Authors:** Yasuhiko Murata, Takako Yasuda, Tomomi Watanabe-Asaka, Shoji Oda, Akiko Mantoku, Kazuhiro Takeyama, Masahiro Chatani, Akira Kudo, Satoko Uchida, Hiromi Suzuki, Fumiaki Tanigaki, Masaki Shirakawa, Koichi Fujisawa, Yoshihiko Hamamoto, Shuji Terai, Hiroshi Mitani

**Affiliations:** 1 Department of Radiation Biology, Graduate School of Medicine, Tohoku University, Miyagi, Japan; 2 Department of Integrated Biosciences, Graduate School of Frontier Sciences, The University of Tokyo, Chiba, Japan; 3 Department of Biological Information, Graduated School of Bioscience and Biotechnology, Tokyo Institute of Technology, Kanagawa, Japan; 4 Japan Space Forum, Tokyo, Japan; 5 Japan Aerospace Exploration Agency, Ibaraki, Japan; 6 Department of Gastroenterology and Hepatology, Yamaguchi University Graduate School of Medicine, Yamaguchi, Japan; 7 Department of Biomolecular Engineering, Graduate School of Medicine, Yamaguchi University, Yamaguchi, Japan; 8 Division of Gastroenterology and Hepatology, Graduate School of Medical and Dental Sciences, Niigata University, Niigata, Japan; The Ohio State University, UNITED STATES

## Abstract

To understand how humans adapt to the space environment, many experiments can be conducted on astronauts as they work aboard the Space Shuttle or the International Space Station (ISS). We also need animal experiments that can apply to human models and help prevent or solve the health issues we face in space travel. The Japanese medaka (*Oryzias latipes*) is a suitable model fish for studying space adaptation as evidenced by adults of the species having mated successfully in space during 15 days of flight during the second International Microgravity Laboratory mission in 1994. The eggs laid by the fish developed normally and hatched as juveniles in space. In 2012, another space experiment (“Medaka Osteoclast”) was conducted. Six-week-old male and female Japanese medaka (Cab strain osteoblast transgenic fish) were maintained in the Aquatic Habitat system for two months in the ISS. Fish of the same strain and age were used as the ground controls. Six fish were fixed with paraformaldehyde or kept in RNA stabilization reagent (n = 4) and dissected for tissue sampling after being returned to the ground, so that several principal investigators working on the project could share samples. Histology indicated no significant changes except in the ovary. However, the RNA-seq analysis of 5345 genes from six tissues revealed highly tissue-specific space responsiveness after a two-month stay in the ISS. Similar responsiveness was observed among the brain and eye, ovary and testis, and the liver and intestine. Among these six tissues, the intestine showed the highest space response with 10 genes categorized as oxidation–reduction processes (gene ontogeny term GO:0055114), and the expression levels of choriogenin precursor genes were suppressed in the ovary. Eleven genes including *klf9*, *klf13*, *odc1*, *hsp70* and *hif3a* were upregulated in more than four of the tissues examined, thus suggesting common immunoregulatory and stress responses during space adaptation.

## Introduction

Space radiation and microgravity are major environmental stressors to humans in space, and the physiological changes induced by those stressors are the main health concerns during space adaptation [1]. How microgravity modifies radiation effects is an important question that must influence our understanding of the hazards encountered by astronauts during prolonged space travel.

To understand how organisms adapt to the space environment, many experiments can be conducted on humans while they work aboard the Space Shuttle or the International Space Station (ISS) [2, 3]. However, various kinds of animal experiments relevant to human physiology are needed to help prevent or solve the health problems astronauts might face. The effects of spaceflight and simulated microgravity on various tissues and systems of the human body have been well documented using animal models for the skeletal system [4–6], brain [7, 8], immune system [9–13], ovary [14], and testis [15, 16]. Several transcriptomic analyses after spaceflight have also been conducted, suggesting that there could be decreases in the expression levels of genes involved in oxidative stress, and in the levels of key proteins involved in mitochondrial fatty acid metabolism [17]. In contrast, the expression levels of genes encoding proteins involved in various cellular defense mechanisms including antioxidation showed increases [18–22]. However, most of these studies concerned alterations in gene expression levels after two weeks of adaptation or less, and no inducing factors could be identified.

In addition to mammals, lower vertebrate such as amphibians have thus far been used for space experiments as model animals. In particular, as the female newt can lay eggs a few months after natural breeding with sperm in the cloacal pelvic glands, the impact of using newt in the space environment on morphogenesis has been verified [23]. Regarding the immune system, genes related to the immune system in mammalians are conserved in amphibians, and a reduction of IgM amounts and alteration of its configuration in the space environment have been reported, suggesting that the immune system may be affected in the space environment [13, 24].

Japanese medaka is a vertebrate fish species commonly used for scientific research because they are smaller than zebrafish, can live in a much smaller area, and consume less food, water, and oxygen [25]. It is a suitable model fish for studying space adaptation, as evidenced by adult fish having mated successfully during 15 days of flight in space in the second International Microgravity Laboratory (IML-2) mission in 1994. The eggs laid by the fish developed normally, and hatched as fry (juveniles) in space [26]. Unlike terrestrial animals including mammalian species that require relatively large volumes of blood (ca. 10% of body mass) at high arterial pressure (ca. 100 mmHg) under normal gravity, teleost fish circulate a small volume of blood (3–4% of body mass) at low blood pressure (20–30 mmHg) in the relatively gravity free aquatic environment [27]. Therefore, fish can be used as the experimental counterparts of mouse or rat models to understand space adaptation mechanisms as fish are less dependent on gravity [28].

In 2012, another space experiment on the effect of microgravity on osteoclasts and analysis of the gravity-sensing system in Japanese medaka (“Medaka Osteoclast”) were conducted (http://www.nasa.gov/mission_pages/station/research/experiments/984.html). In the present study, male and female fish (six-weeks old at launch) were cultured in the Aquatic Habitat (AQH) system [17] for two months in the ISS. Six fish were fixed with 4% paraformaldehyde (PFA) or kept in RNA stabilization reagent (n = 4) and dissected for tissue sampling after being returned to the ground, so that several principal investigators (PIs) working on this project could share samples. Here we report on histological and transcriptomic analyses using six isolated tissues (brain, eye, ovary, testis, liver, and intestine) after 56–60 days of exposure to the space environment.

## Materials and Methods

### Experimental Design and Animal Care

The protocol utilized in the study was authorized by the Committee on Animal Care and Use at the Japan Aerospace Exploration Agency (No. 011–014A) and by the American Association or Laboratory Animal Science at the National Aeronautics and Space Administration (NAS-12–003-Y1). The experimental procedures were conducted in accordance with the guidelines for the Care and Use of Laboratory Animals of the University of Tokyo and Tokyo Institute of Technology.

The spaceflight experiments were conducted using the F1 fish of two closed colonies; Japanese medaka wild type Cab and Cab strain transgenic fish (TRAP:GFP, Osterix:DsRed), which were originally kept in the fish facility at the Tokyo Institute of Technology [29, 30]. At this age, the sex of immature fish cannot be determined. The F1 fish (16 mm in length, six-weeks old at launch, and n = 8 in each of two aquaria) were transferred from the “Fish Carrier” to the two aquaria of the AQH, which is installed in a small powered multipurpose payload rack and housed in the Japanese Experiment Module (Kibo) on the ISS [17]. These transgenic fish were cultured as the spaceflight group (SF) in the AQH from October 26 to December 24, 2012, and utilized for the “Medaka Osteoclast” space experiment. Three out of eight fish in each aquarium were removed 14 days after incubation for “Medaka Osteoclast.” The other fish were maintained in the AQH system for two months in the ISS. This habitat provides automatic feeding for the fish, an air–water interface, temperature control, and a specimen sampling mechanism, and enables the monitoring of behavior 24 hours a day [31]. There are two chambers for habitation, each holding about 700 ml of water. Light-emitting diodes were used to simulate 14-h day and 10-h night cycles. The animals and tissues we used in this study were shared with the “Medaka Osteoclast” experiment.

### Sample Preparation

In this study, no abnormalities in swimming and feeding behavior were observed in the SF group or GC group, and we used all the samples following the experiment. Sample fixations were performed between 8:20–14:00 without feeding in the morning on the sampling day. Six fish were sampled for histological analysis at 56 days of flight in the ISS, and four were used for RNA-seq analysis at 60 days. Fish were transferred using the “Fish Catcher” (a special large pipette used to remove fish from each aquarium for transfer to the “Fish Fixation Apparatus,” which is a chemical fixation kit constructed from triple-layered plastic bags, with 6 ml of breeding water for histology and 5 ml for RNA-seq analysis of each fish. For histology, three fish were transferred to this bag with approx. 18 ml of breeding water. The fish were treated with approx. 0.018% of anesthetic MS222 (tricaine methanesulfonate) solution by mixing 4 ml of 1 g/L MS222 to 18 ml of breeding water. Then the whole bodies of fish were fixed in approx. 4% paraformaldehyde in 0.1 M cacodylate acid buffer (pH 7.4) solution by mixing 20 ml of 8.6% paraformaldehyde in 0.215 M cacodylate acid buffer solution to 22 ml of 0.018% anesthetic solution, and kept at 2°C. After three or four days of fixation, fixative was replaced by 0.1 M cacodylate acid buffer solution (pH 7.4) and kept at 2°C for later analysis.

For the RNA-seq analysis, four fish were used and two each were stacked into a bag with 320 mg/L of anesthetic MS222 by breaking the pouch containing 5 ml of 700 mg/L MS222 and mixing the solution. Then anesthetics were removed by sponge absorption, replaced by the RNA stabilization reagent RNA*later*® (Thermo Fisher Scientific Inc., Waltham, MA, USA), and kept at –95°C.

After the spaceflight experiment, a group of fish of the same strain and age were housed in an AQH for two months at Tsukuba Space Center in Ibaraki, Japan as the ground control (GC) group. As space experiments were conducted under several limited conditions, it was necessary to perform ground control after the space experiment, in order to follow the same conditions as those used in the space experiment. GC samples were treated the same way as for the SF group, with PFA fixation and RNA storage being conducted at an interval of four days (56 and 60 days after breeding). The SF and GC experiments were only conducted once. All samples were used for the subsequent experiments. Fixed samples in RNAlater^®^, which became hard, were carefully dissected on a 90-mm dish (As One, GD 90–15) using noyes spring scissors (Nisshin Em Co., Ltd. Tokyo, 5 m/m), forceps (Dumont 5-Inox-H), and ring forceps (Napox A-26) under a microscope. The brain and eyes were separated from the head region, and the liver, intestine, testis, and ovary were separated by an abdominal operation. Fixed samples from all six siblings were used for tissue sharing with other PIs.

### Histology

**Fig 1:**
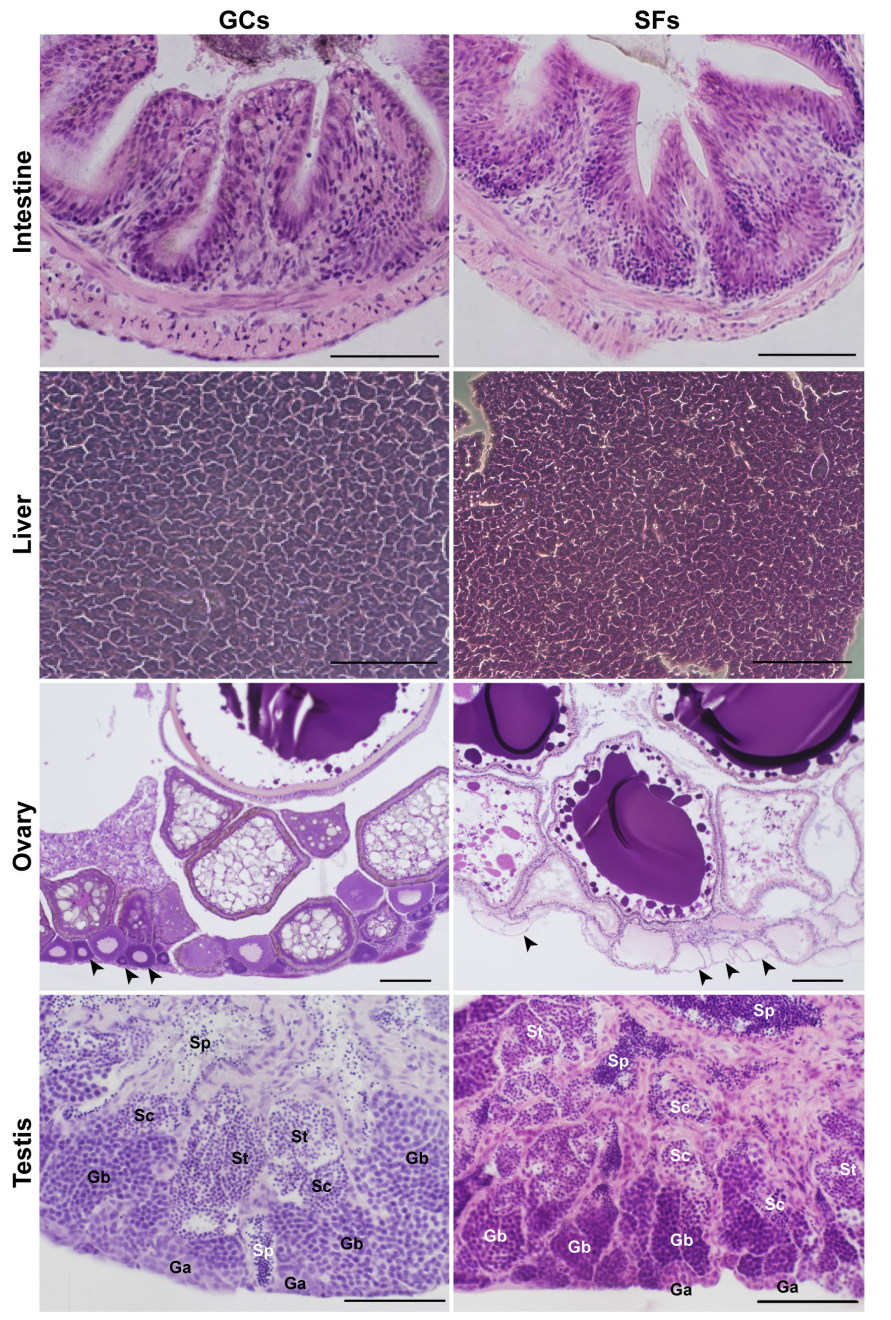
No morphological alteration appeared except in the ovary after two months in the International Space Station. H&E staining of tissues in the ground control (GC) and spaceflight (SF) groups. Histology is shown of the caudal part of the intestine, and the liver, ovary, and testis. Scale bars = 100, 200, 50, and 50 μm for images of the intestine, liver, ovary, and testis, respectively. Arrowheads show the previtellogenic stage oocytes. Ga: cyst of type A spermatogonia, Gb: cyst of type B spermatogonia, Sc: cyst of spermatocyte, Sp: sperm mass, St: cyst of spermatocyte

Whole body fixation with PFA was performed in the GC and SF groups described above, and the samples used for conventional histology were placed in Davidson’s fixative (22 ml of 37–40% formaldehyde, 33 ml ethanol, 11.5 ml glacial acetic acid, and 33.5 ml distilled H_2_O in 100 ml Davidson’s fixative) after dissecting out the intestine. The ovary, testis, liver, and intestine were used in this study. The intestine was separated into four portions longitudinally. Epithelia were not fixed well except for the most caudal part of the intestine in both the SF and GC groups as compared with conventional Davidson’s fixation after dissection (Fig 1 and Supporting Information S1 Fig). Testicular type A spermatogonia was not fixed well and shrank after fixation in both the SF and GC groups, as compared with conventional Davidson’s fixation (Fig 1 and S2 Fig). Samples were dehydrated through an ethanol series and then embedded in Technovit resin (ovary, testis, and intestine) or paraffin wax (liver). Sections 7-mm thick were then stained with hematoxylin and eosin (H&E) to analyze morphological alteration [32].

### RNA-Seq

To compare the gene expression pattern between SF and GC, fixed samples were dissected under a microscope and total RNA was isolated from 2–4 siblings using TRIzol® reagent (Thermo Fisher Scientific Inc.) from the brain, eye, liver, intestine, testis, and ovary. The intestine was separated into four portions longitudinally and high quality RNA could only be obtained from most caudal part of the intestine. RNA quality was evaluated using an Agilent Bioanalyzer 2100 total RNA Nano series II chip (Agilent Technologies Inc., Santa Clara, CA, USA) and total RNA was pooled from the same tissue as a single sample in this study. The RIN numbers of the testis, ovary, liver, brain, eye, and intestine in the SF samples were 7.1, 7.3, 7.9, 8.1, 8.6, and 7.1, respectively. The RIN numbers of the testis, ovary, liver, brain, eye, and intestine in the GC samples were 7.5, 7.7, 8.0, 7.8, 8.1, and 8.7, respectively. RNA-seq analysis was conducted commercially by the Takara *de novo* RNA-seq service (Takara Bio Inc., Shiga, Japan). Transcriptome libraries were prepared from total RNA using Illumina TruSeq Stranded RNA Sample Preparation kits (Illumina Inc., San Diego, CA, USA). Libraries were sequenced by a single read length of 100 nucleotides on the Illumina Hi-Seq2500 instrument as per the manufacturer’s recommendations. The resulting reads were trimmed of low quality nucleotides and aligned against 24662 cDNA sequences predicted by Ensembl’s *Oryzias latipes* Genebuild (version 56, www.ensembl.org). Gene expression levels were determined by measuring the sum of the reads per kilobase per million mapped read (RPKM) values of each exon. Alignment counts were normalized for transcript lengths and total aligned RPKM values. To acquire more accurate results, we filtered out the data for samples when the estimated RPKM values in the SF or GC sample were < 1.0 or when the read numbers were < 5. Table 1 summarizes the numbers of genes examined in each tissue. Fisher’s exact test was conducted to compare the expression ratio between the intestine and the other tissues. The mRNA-Seq data from our experiments have been submitted to the DNA Data Bank of Japan (DDBJ) (http://www.ddbj.nig.ac.jp/) under accession no. DRA003542.

**Table 1:** Total numbers of genes and numbers of genes with at least a twofold increase or decrease in gene expression in SF tissues.

	Brain	Eyes	Ovary	Testis	Liver	Intestine
Twofold increase	91*	112*	1562*	731*	378*	2279
Twofold decrease	45*	84*	240*	249*	648*	492
Total numbers of genes	13528	14459	10836	11887	7385	9905

Asterisks indicated p < 0.0001 as compared to the intestine by Fisher’s exact test. SF: spaceflight

The gene expression ratio between the SF and GC tissues was defined as Space Responsiveness (SR):

$$SR={\text{log}}_{2}({RPKM}_{SFs}\;/\;{RPKM}_{GCs})$$

To compare gene responsiveness to the space environment among tissues, we selected 5,345 genes showing a minimum expression level as RPKM ≥ 1 and a number of unique reads with RPKM ≥ 5 in all six tissues.

Total Space Responsiveness (||TSR||) was defined as:

$$∥TSR∥=\sqrt{SR_{brain}{}^{2}+SR_{eye}{}^{2}+SR_{liver}{}^{2}+SR_{intestine}{}^{2}+SR_{ovary}{}^{2}+SR_{testis}{}^{2}}$$

### Gene Ontology (GO) Analysis

All exons from the transcript GO identity numbers (IDs) of a gene ID were integrated, and the integrated exons were identified as gene positions of the gene ID. When exon lengths differed between transcript IDs for the same gene ID, the longer exon was selected. All sequenced reads mapped to the region of integrated exons used Bowtie (version 0.12.9) for calculating gene expression levels [33]. The GO IDs used in this analysis were obtained using the following steps. First, human Ensembl gene IDs that were orthologues for the Japanese medaka Ensembl gene IDs were obtained from the dataset of *Oryzias latipes* genes of the Hd-rR strain using BioMart of the Ensembl database (http://www.ensembl.org/index.html). Next, Entrez gene IDs associated with human Ensembl gene IDs were extracted from gene2ensembl (ftp://ftp.ncbi.nlm.nih.gov/gene/DATA/gene2ensembl.gz) in the NCBI database (http://www.ncbi.nlm.nih.gov/).

The GO IDs associated with the Entrez gene IDs were finally extracted from gene2go (ftp://ftp.ncbi.nlm.nih.gov/gene/DATA/gene2go.gz) in the NCBI database. The GO IDs were counted and organized using customized Perl scripts. For the GO IDs of each of the gene groups classified by gene expression levels, hypergeometric distribution analysis was conducted using R software (version 3.1.2, http://cran.r-project.org/doc/manuals/r-patched/R-admin.html). The threshold of *p*-values in the analysis was set at 0.05. False discovery rates were calculated using the Benjamini–Hochberg method [34], and the threshold in the analysis was set at 0.05.

Hierarchal cluster analysis was conducted with tree clustering using Cluster 3.0, an enhanced version of Cluster originally developed by Michael Eisen at Stanford University (http://bonsai.hgc.jp/~mdehoon/software/cluster/manual/index.html), and TreeView programs, which allow interactive graphical analysis of the results from Cluster 3.0 (http://jtreeview.sourceforge.net/).

## Results

### Histology showed slight disruption in oogenesis after two months of spaceflight.

To find morphological alteration in tissues after two month in the space environment, we analyzed the paraffin embedded sections of several tissues. Tissue structures were no different between the SF (n = 4) and GC (n = 5) groups in the caudal part of the intestine (Fig 1). Using H&E staining of the liver, cellular abnormalities such as vacuolization, fibrosis, the appearance of spindle-like cells with pyknotic nuclei, and malignant transformation were not observed in either group (n = 5 each). In the ovary, we found the depletion of previtellogenic stage oocytes in all SF samples (n = 3), but not in the GC samples (n = 2) (Fig 1 and S3 Fig). This suggested that oogenesis was slightly disrupted, even though body growth and maturation were not delayed, and mating behavior occurred in the space environment every day from the 27^th^ day after launch—timing similar to that in the GC group (Chatani et al., submitted elsewhere). However, the fixation conditions were obviously different in the space environment, which lacked circulation as compared with the ground controls. In the testis, although cysts of type A spermatogonia were not well fixed and could not be identified, other structures such as Gb cysts, spermatocytes, spermatids, and spermatozoa were similar between the GC (n = 3) and SF (n = 2) groups, suggesting that normal spermatogenesis was maintained during spaceflight.

### Transcriptomic analysis reveals the highest level of spaceflight-induced changes in the intestine.

Next, RNA-seq analysis was conducted to find alteration in the gene expression during two months of spaceflight. S4 Fig shows the distribution of the number of genes responsive to SFs among different tissues. Table 1 summarizes the numbers of genes showing more than a twofold difference in expression level in the SF tissues. The numbers of responsive genes were clearly tissue-dependent (S1–S12 Tables). A few genes in the brain and eyes responded to the space environment. On the other hand, more than 2,500 genes changed their expression levels in the caudal part of the intestine, suggesting that this was the most responsive tissue (S5 and S6 Tables). Moreover, many genes were downregulated in the liver without accompanying histological abnormalities (S7 and S8 Tables).

### GO analysis presents different gene expression patterns among tissues in the space environment.

Spaceflight-induced transcriptome alterations (with more than two-fold differences) in various GO categories were detected in the brain, eye and intestine (Tables 2–4). Gene expression was significantly increased in 11 categories in the brain. Although only a few genes were space-responsive in the eyes, in the space environment GO:0071889 (14-3-3 protein binding) was significantly enhanced in both the brain and eyes, while GO:0030049 (muscle filament sliding) was significantly enhanced in the brain. In the intestine, gene distribution was significantly increased in 12 GO categories including GO:0000502 (proteasome complex), GO:0002479 (antigen processing and presentation of exogenous peptide antigens via MHC class I molecules), GO:0006915 (apoptotic processes), GO:0000278 (mitotic cell cycle), and GO:0006977 (DNA damage response). However, no significant alterations in gene expression levels (i.e., more than a twofold difference) were detected in the ovary. Because histological changes in the ovary suggested that the growth of oocytes was disturbed, several genes related to oogenesis were extracted from the database. The expression levels of zona pellucida glycoprotein genes were not affected; however, the space environment suppressed choriogenin precursor gene expression in the ovary (Table 5).

**Table 2:** GO analysis of genes differentially expressed in the SF brain.

GO ID	GO term	Upregulation /Downregulation	Number of genes with upregulation / downregulation in GO category	Number of genes in GO category	p-value
GO:0071889	14-3-3 protein binding	Up	3	18	2.23E–04
GO:0030049	Muscle filament sliding	Down	6	27	2.69E–10
GO:0005861	Troponin complex	Down	3	6	6.83E–07
GO:0003009	Skeletal muscle contraction	Down	3	10	4.06E–06
GO:0030017	Sarcomere	Down	3	24	6.63E–05
GO:0008307	Structural constituents of muscle	Down	3	25	7.52E–05
GO:0006096	Glycolytic processes	Down	3	35	2.09E–04
GO:0006094	Gluconeogenesis	Down	3	38	2.67E–04
GO:0008092	Cytoskeletal protein binding	Down	3	39	2.89E–04
GO:0006600	Creatine metabolic process	Down	2	10	4.79E–04
GO:0003779	Actin binding	Down	5	217	7.35E–04
GO:0006936	Muscle contraction	Down	3	56	8.43E–04

The table lists the GO annotation terms for 91 genes that showed at least a twofold increase (S1 Table) or 45 genes that showed at least a twofold decrease in expression levels (S2 Table). The GO annotation terms show their statistically acceptable p-values (<0.05). SF: spaceflight

**Table 3:** GO terms for genes differentially expressed in the SF eye.

GO ID	GO term	Upregulation / Downregulation	Number of genes with upregulation / downregulation in GO category	Number of genes in GO category	p-value
GO:0071889	14-3-3 protein binding	Up	4	20	1.50E–05
GO:0001076	RNA polymerase II transcription factor binding activity	Down	3	10	2.20E–05
GO:0006953	Acute phase response	Down	3	15	8.18E–05

The table lists the GO annotation terms for 112 genes in S3 Table that showed at least a twofold increase in gene expression and 84 genes in S4 Table that showed at least a twofold decrease in gene expression. The GO annotation terms show their statistically acceptable p-values (<0.05). SF: spaceflight

**Table 4:** GO terms for genes with more than a twofold expression in the SF intestine.

GO ID	GO term	Number of genes with upregulation / downregulation in GO category	Number of genes in GO category	p-value
GO:0005829	Cytosol	470	1592	2.75E–11
GO:0000502	Proteasome complex	21	31	1.41E–07
GO:0070062	Extracellular vesicular exosome	373	1346	8.51E–06
GO:0002479	Antigen processing and presentation of exogenous peptide antigens via MHC class I molecules	23	43	1.36E–05
GO:0002474	Antigen processing and presentation of exogenous peptide antigens via MHC class I molecules	28	57	1.38E–05
GO:0042590	Antigen processing and presentation of exogenous peptide antigens via MHC class I molecules	24	46	1.55E–05
GO:0006915	Apoptotic processes	110	337	2.49E–05
GO:0000278	Mitotic cell cycle	77	221	3.63E–05
GO:0051084	*De novo* posttranslational protein folding	15	24	3.89E–05
GO:0006977	DNA damage response	20	38	6.74E–05

The table lists the GO annotation terms for 2279 genes in S5 Table that showed at least a twofold increase in gene expression. There were no GO annotation terms for 492 genes in S6 Table that showed at least a twofold decrease in gene expression. The GO annotation terms show their statistically acceptable p-values (<0.05). MHC: major histocompatiblity complex, SF: spaceflight

**Table 5:** Expression levels and SR of genes associated with egg growth in the ovary of the GC and SF groups.

Gene symbol	Gene name	Ensembl *Oryzias latipes* gene ID	RPKM (GC)	RPKM (SF)	SR
1-sf	L-SF precursor	010134	7.23	2.22	–1.70
ZP2(1 of 3)	Zona pellucida glycoprotein 2	006621	457.84	355.68	–0.36
ZP2(2 of 3)	Si: dkeyp-50f7.2	012471	2068.80	2059.62	–0.01
ZP2 (3 of 3)	Si: ch211-223m11.2	012527	1223.85	1321.62	0.11
zp3b	Zona pellucida glycoprotein 3b	016064	678.32	710.87	0.07
	Choriogenin H minor	010086	1.61	0.65	–1.31
	Choriogenin H precursor	010880	7.42	2.61	–1.51
	ZPB domain-containing protein	016580	1824.70	1722.53	–0.08
	ZPC domain containing protein 3	009867	907.89	876.16	–0.05

Gene IDs and their descriptions (http://www.ensembl.org/Oryzias_latipes/Info/Index)

Acc: account, GC: ground control, RPKM: Reads Per Killobases per Million, SF: spaceflight, Si: gene represented by annotated genomic sequence from the Sanger Institute, SR: space responsiveness = Log_2_(RPKM of SFs/RPKM of GCs), ZFIN: Zebrafish Model Organism Database

### Oxidative stress related genes were upregulated specifically in the intestine during spaceflight.

**Fig 2:**
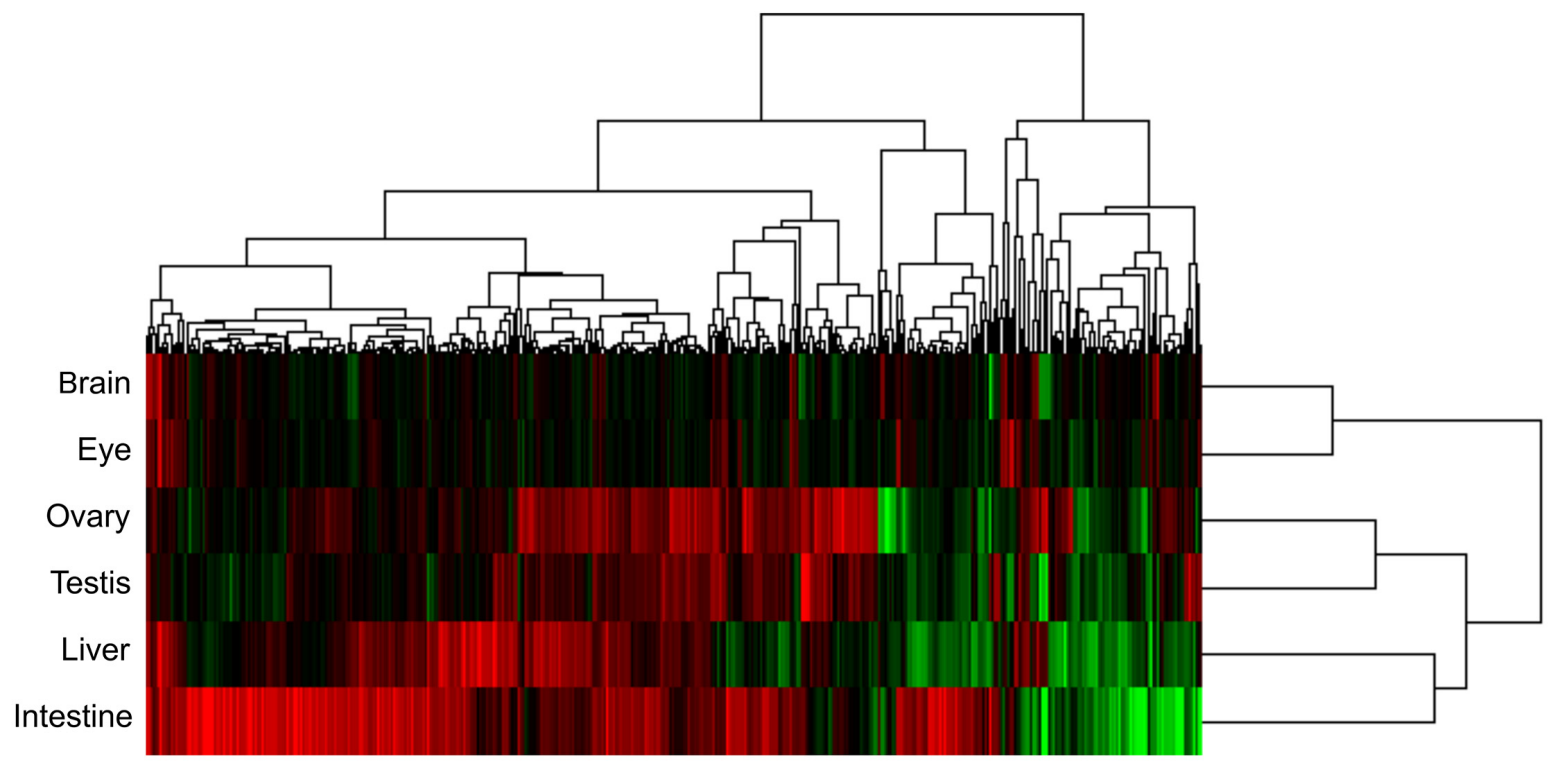
Hierarchical cluster analysis of gene expression levels derived by conditional tree clustering. The top 400 genes ranked by Total Space Responsiveness (||TSR||) were analyzed using Cluster version 3.0. Red and green indicate increases and decreased expression levels of each gene in response to spaceflight, respectively.

The top 400 genes ranked by ||TSR|| were analyzed using Cluster version 3.0 (Fig 2). Most of the genes showed high tissue specificity. We identified five clusters based on tissue dependence of the SR patterns, but no preferential distribution for specific GO categories was detected in any clusters. Similar responsiveness was observed between the brain and eye, the ovary and testis, and the liver and intestine. Eleven genes including *klf9*, *klf13*, *odc1*, *hsp70*.*3* and *hif1a1*, which was annotated as *hif3a* in the human GO analysis, were upregulated in more than four of the tissues examined, thus suggesting common immunoregulatory and stress responses in multiple tissues during space adaptation for two months or less (Table 6). Moreover, among the 47 intestine-specific genes with SFs upregulated or downregulated, 10 were categorized as GO:0055114 (oxidation-reduction processes). There were seven upregulated genes (prdx1, GSTO1, cyba, steap3, pgd, arf2, and TXNDC2) and three downregulated genes (Scdb, HSD11B1L, and CDO1L). This suggests that the caudal part of the intestine was highly sensitive to oxidation-reduction stress in the space environment (Table 7).

**Table 6:** Genes with high SR values (>1 or <-1) in more than four tissues,

Gene symbol	Gene name	Ensembl *Oryzias latipes* Gene ID	SR (Brain)	SR (Eye)	SR (Ovary)	SR (Testis)	SR (Liver)	SR (Intestine)	||Z||
dusp1	Dual specificity phosphatase 1	006559	1.31*	1.12*	1.18*	0.40	1.02*	0.81	2.49
hif1a1	Hypoxia-inducible factor 1, alpha subunit-like	002004	0.31	0.28	1.25*	1.42*	1.49*	1.30*	2.76
hsp70.3	Heat shock cognate 70-kd protein, tandem duplicate 3	000233	2.06*	2.14*	–0.16	–0.34	2.57*	1.47*	4.21
keap1a	Kelch-like ECH-associated protein 1a	013993	–0.13	–0.41	–1.51*	–1.52*	–1.00*	–1.11*	2.64
klf9	Kruppel-like factor 9	010690	2.69*	2.72*	0.59	0.40	4.48*	4.34*	7.35
klf13	Kruppel-like factor 13	007542	2.06*	1.25*	0.35	1.35*	1.88*	2.56*	4.22
mat2ab	Methionine adenosyltransferase II, alpha b	006298	0.23	0.10	1.44*	1.26*	2.36*	1.22*	3.28
nrld4b	Nuclear receptor subfamily 1, group D, member 4b	015399	–0.02	1.29*	1.38*	1.68*	0.34	1.16*	2.80
odc1	Ornithine decarboxylase 1	017758	–0.43	–0.23	2.04*	1.04*	2.44*	3.07*	4.57
tmem39a	Transmembrane protein 39A	003717	0.21	–0.11	1.34*	1.14*	1.98*	1.05*	2.86
usp10	Ubiquitin-specific peptidase 10	007919	–0.36	–0.24	1.19*	1.07*	1.46*	1.06*	2.45
zfp36	Zgc: 162730	008378	1.76*	1.55*	1.28*	0.31	–0.27	1.84*	3.27

Acc: account, RPKM: Reads Per Killobases per Million, SR: space responsiveness = Log2(RPKM of SFs/RPKM of GCs), ||Z||: absolute z score, ZFIN: Zebrafish Model Organism Database. Asterisks indicate high (≥1) or low (≤-1) SR value.

**Table 7:** Space responsive genes in the intestine–categorized as GO:0055114.

Gene symbol	Gene name	Ensembl Oryzias latipes Gene ID	SR (Intestine)	SR (Brain)	SR (Eye)	SR (Ovary)	SR (Testis)	SR (Liver)	||Z||
prdx1	Peroxiredoxin 1	010533	4.66	–0.15	0.07	–0.42	–0.36	0.53	4.72
GSTO1	Glutathione S-transferase omega 1	006201	3.43	–0.27	–0.05	–0.32	–0.64	–0.07	3.52
cyba	Cytochrome b-245, alpha polypeptide	009360	3.45	–0.60	–0.46	–1.29	0.11	–0.48	3.79
steap3	STEAP family member 3, metalloreductase	004437	3.43	–0.20	0.11	–0.06	–0.20	0.17	3.45
pgd	Phosphogluconate hydrogenase	005212	3.02	–0.19	–0.16	0.45	0.28	0.21	3.08
arf2	ADP-ribosylation factor 2	014113	2.79	–0.13	0.03	0.42	0.17	0.02	2.83
txndc2	Thioredoxin domain-containing 2	007575	2.52	0.08	0.17	0.08	–0.27	–0.16	2.55
scdb	Stearoyl-CoA desaturase b	006839	–2.76	–0.26	–0.17	1.01	0.80	0.47	3.10
hsd11b1l	11-beta-hydroxysteroid dehydrogenase type 3	004562	–2.88	–0.02	–0.39	–0.77	0.31	–0.69	3.10
cdo1	Cysteine dioxygenase type 1	003390	–5.83	0.14	0.72	–2.12	–0.21	–0.67	6.28

Genes are aligned in descending order of SR values in the intestine. Acc: account, HGNC: Human Genome Organisation Gene Nomenclature Committee, RPKM: Reads Per Killobases per Million, Si: gene represented by annotated genomic sequence from the Sanger Institute, SR: space responsiveness = Log2(RPKM of SFs/RPKM of GCs), ||Z||: absolute z score, ZFIN: Zebrafish Model Organism Database

## Discussion

Here we conducted both histological and transcriptomic analyses using Japanese medaka fish adapted to the space environment for two months, and performed sampling while still in the ISS. The RNA-seq technique allows quantitative measurement of transcriptomes in a high-throughput manner with a high dynamic range of quantitative measurement.

The tissue structures of the liver, ovary, and the most caudal part of the intestine were well retained by chemical fixation in the ISS, and high quality RNA could only be obtained from these tissues. However, the rostral part of the intestine and type A spermatogonia in the testis did not fix well. In the ovary, there was depletion of previtellogenic stage oocytes, and the expression levels of choriogenin precursor genes were suppressed only in the ovary. These results suggest that oogenesis could be suppressed, such as in the quality of eggs after two months in the space environment, unless there was no delay in mating behavior. In a mouse model of spaceflight, the presence of developing mouse follicles of all stages, together with the corpora lutea in all three treatment groups, two sets of control animals, and a single set of flight animals, indicated that no significant gross morphological changes occurred in ovarian tissues when exposed for up to 13 days [14]. It is known that maturation of the medaka ovary begins one week after hatching [35]. We analyzed fish that were launched at six weeks of age and found changes in the ovary after two months. The gene expression was altered even though the appearance of mating behavior suggested that a very slight change had occurred in two months, and that the longer the duration in the space environment, such as being born in the ISS, the more severe the phenotype may appear. Moreover, our results suggest that fish grown in the space environment from this immature stage would show more severely affected phenotypes relative to the numbers of oocytes and oogenesis. Several factors could account for alterations in the medaka ovary after a two-month stay in the ISS, such as prolonged exposure to low-dose space radiation and endocrine changes under microgravity.

The transcriptomic analysis showed large differences in space responsiveness among the tissues, and that most of the changes were highly tissue-dependent. Some stress-related GO gene profiles were altered after space adaptation; specifically those encoding 14-3-3 protein binding (GO:0071889) in the brain and eyes; those encoding antigen processing and presentation of exogenous peptide antigens via MHC class I molecules (GO:0002479); those encoding apoptotic processes (GO:0006915); and DNA damage responses (GO:0006977) in the intestine were all increased. In addition to the GO analysis, we also identified 11 upregulated genes and one downregulated gene showing multi-tissue responses related to cell proliferation and oxygen-related stress responses. The intestine proved more highly sensitive to the space environment than the other tissues at the single gene expression level. Moreover, 10 genes in the category of oxidation–reduction processes (GO:0055114) showed intestinal specific responses in the SF group. Reductions in liver, spleen, and thymus masses were observed, and many of the genes responsible for scavenging reactive oxygen species were upregulated in C57BL/6NTac mice after spaceflight during a 13-day Space Shuttle mission (STS-118) [36]. The effects of three-month exposure to the space environment using the Mice Drawer System on the expression of genes and proteins in the mouse brain were reported [8, 37]. The biological functions of the upregulated genes were related to immune response, metabolic processes, and/or inflammatory response; the downregulated functions were related to catalytic and oxidoreductase enzyme activities. The present study supported previous findings in that the genes involved in encoding stress factors, such as immune and inflammatory responses, were upregulated or downregulated in the space environment.

Moreover, throughout the evolution of the cellular immune system, members of the MHC class I superfamily have served as indicators of intracellular stress response [38]. Before adaptive immunity, class I-like molecules likely reported the stress status of cells to primordial migratory leukocytes. In the ER, chaperones guide the assembly of MHC class I α chain, β2-microglobulin, and peptides. Stress stimuli such as heat shock, hypoxia, viral replication, abnormal proteins, starvation or transformation ultimately lead to an accumulation of unfolded or misfolded proteins in the lumen of the ER. The tissue-specific ER responses may be activated through intracellular signal transduction pathways during space adaptation. Although it is important to replicate the present experiment, these findings could be utilized as markers of common space adaptations in vertebrates ranging from fish to humans. Moreover, generating transgenic fish that express the candidate marker genes found in this study could be a useful tool for evaluating the biological effects of the space environment.

There were no alterations in the expression level of genes involved in metabolism and digestive enzymes, suggesting that the major processes involved in food intake or growth were not altered in the space environment. Previous experiments showed that medaka could mate, and that healthy embryos could hatch and mature after being returned to earth. Those results indicated that this species is a useful model for understanding long-term adaptations to the space environment [26]. The mating behavior that was also observed in this system confirmed the previous experiments (Chatani et al., unpublished results). It was also useful to compare changes in gene expression levels and tissue structures among individual fish under the same culture conditions, as six adult fish could be maintained using the AQH system. This number of animals increases the amount of acquirable data and enhances the statistical significance of the experiments. Thus, it will be important to replicate these experiments.

In this study, the fish were kept over a single generation for two months using the AQH system, in which the breeding of three generations of medaka in the ISS environment is possible [17]. Such a three-generation culture enables the study of mechanisms for adapting to the space environment by observing fish that have been born and raised in space. Moreover, by conducting experiments using space-born fish in this culture system, it will be possible to verify the maturation and maintenance of oocytes in the ovary. Future experimental designs should take into account the overall intricacies of the female reproductive system and its inter-relationship with other physiological systems of the animal.

Epithelial structures, except for the most caudal part of the intestine and Ga cysts in the testis, were not fixed well by whole body fixation with PFA in both the SF and GC groups. Davidson’s fixative is normally used for the medaka testis and intestine [32]. Conversely, the fixation of pharyngeal teeth or muscles is not suitable. In future space experiments, whole body treatment with Davidson’s fixative should be used for histological analyses.

This study focused on particular organs such as the ovary, intestine, testis, liver, brain, and eyes, and analyzed the biological impact of the ISS environment on these organs. We aimed to share samples among PIs in order to evaluate particular tissues. However, the evaluation of blood vessels or alterations of small regions of tissues such as seen in atrophy is difficult when using this method. For such evaluation, it is necessary to make serial sections of the whole body, and the size of Japanese medaka is small enough for that purpose [39]. The analysis of whole body serial sections and RNA expression analysis isolated from each individual organ will reveal physiological responses to the space environment at the molecular, cellular and tissue levels, and demonstrate any variability between individuals. In addition, the preparation of whole body serial sections would make it possible to share the results in the form of a database. Therefore, as the next step in space experimentation, two kinds of experiments will be required: further replicate experiments to evaluate RNA expression levels as biomarkers, and whole body serial sectioning for sharing data among scientists.

## Supporting Information

S1 Fig Degenerative rostral part of the intestine after PFA fixation. Epithelia were not fixed well in the rostral part of the intestine in both the SF and GC groups AS compared with Davidson’s fixative as A conventional method. The caudal part of the intestine was fixed well in all samples. Scale bars = 50 μm.
(PDF)

S2 Fig Type A spamatogonia not fixed well after PFA fixation. Whole body fixation with PFA was performed in the GC and SF groups, and the samples used for conventional histology were placed in Davidson’s fixative. Ga: cyst of type A spermatogonia, Gb: cyst of type B spermatogonia, Sc: cyst of spermatocyte, Sp: sperm mass, St: cyst of spermatocyte. Arrowheads indicate type A spermatogonia, which were clearly observed only in tissues prepared using conventional Davidson’s fixation. Scale bars = 50 μm.
(JPG)

S3 Fig H&E staining of the ovary showing reduction of previtellogenic stage oocytes after two months of space adaptation. Two female fish in the GC group and three in the SF group were fixed with PFA. Fig 1 presents one GC samples and one SF sample, with the rest being shown in this figure. Arrowheads showed the previtellogenic stage oocytes. Scale bars = 50 μm.
(JPG)

S4 Fig Histograms of gene expression ratios in each tissue between the SF and GC groups. Gene expression analyses were conducted for the caudal part of the intestine, and the liver, ovary, testis, brain, and eyes.
(PDF)

S1 Table Genes upregulated in SFs (brain). (XLSX)

S2 Table Genes downregulated in SFs (brain). (XLSX)

S3 Table Genes upregulated in SFs (eye). (XLSX)

S4 Table Genes downregulated in SFs (eye). (XLSX)

S5 Table Genes upregulated in SFs (intestine). (XLSX)

S6 Table Genes downregulated in SFs (intestine). (XLSX)

S7 Table Genes upregulated in SFs (liver). (XLSX)

S8 Table Genes downregulated in SFs (liver). (XLSX)

S9 Table Genes upregulated in SFs (ovary). (XLSX)

S10 Table Genes downregulated in SFs (ovary). (XLSX)

S11 Table Genes upregulated in SFs (testis). (XLSX)

S12 Table Genes downregulated in SFs (testis). (XLSX)
