# Supplementary figures and images for: Histological and Transcriptomic Analysis of Adult Japanese Medaka Sampled Onboard the International Space Station
Source: PLoS One. 2015 Oct 1;10(10):e0138799. doi: 10.1371/journal.pone.0138799 (PMC4591011; doi:10.1371/journal.pone.0138799)

**conventional**

**GCs**

**SFs**

**rostral**

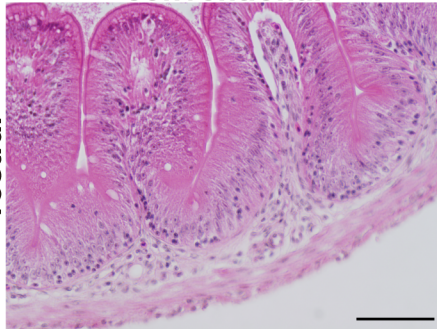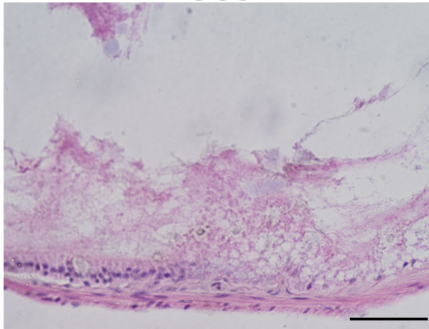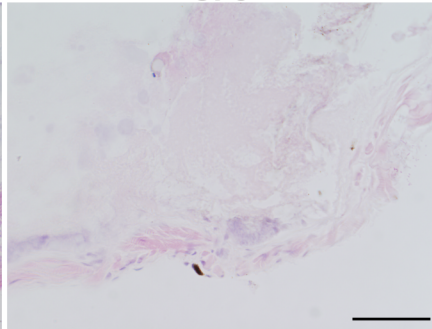

**caudal**

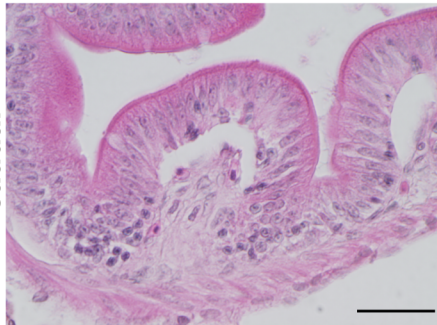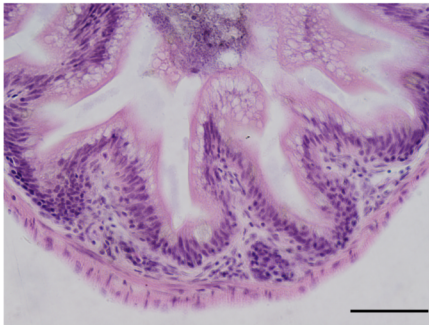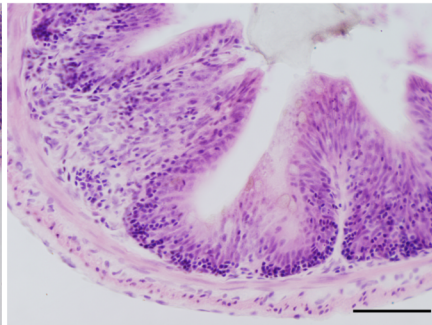

Supplement: S1 Fig — Epithelia were not fixed well in the rostral part of the intestine in both the SF and GC groups AS compared with Davidson’s fixative as A conventional method. The caudal part of the intestine was fixed well in all samples. Scale bars = 50 μm. (PDF) [file pone.0138799.s001.pdf]

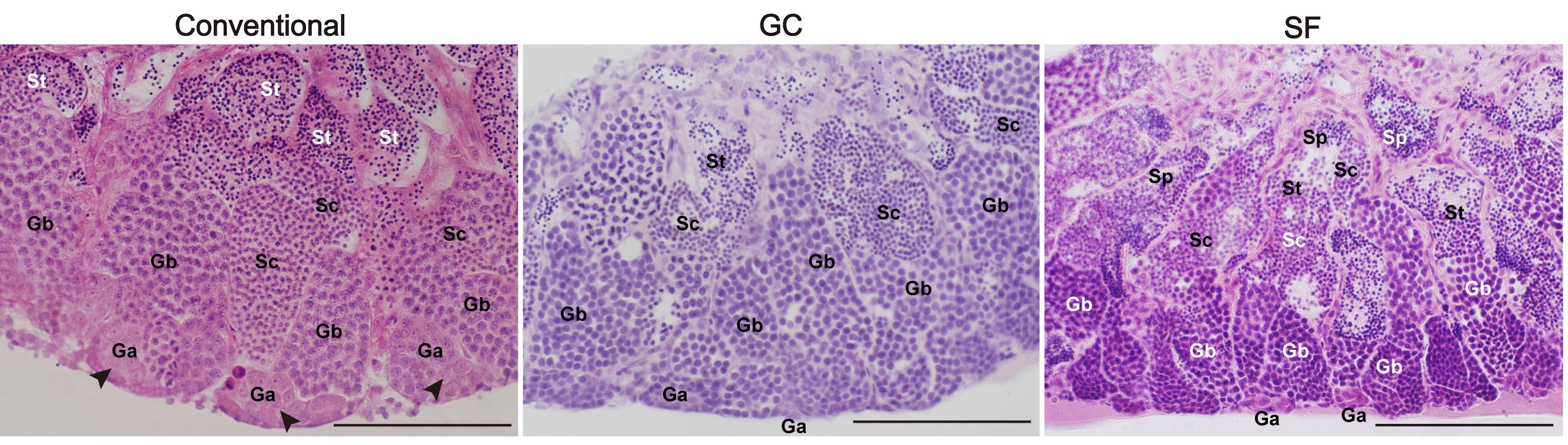

Supplement: S2 Fig — Whole body fixation with PFA was performed in the GC and SF groups, and the samples used for conventional histology were placed in Davidson’s fixative. Ga: cyst of type A spermatogonia, Gb: cyst of type B spermatogonia, Sc: cyst of spermatocyte, Sp: sperm mass, St: cyst of spermatocyte. Arrowheads indicate type A spermatogonia, which were clearly observed only in tissues prepared using conventional Davidson’s fixation. Scale bars = 50 μm. (JPG) [file pone.0138799.s002.jpg]

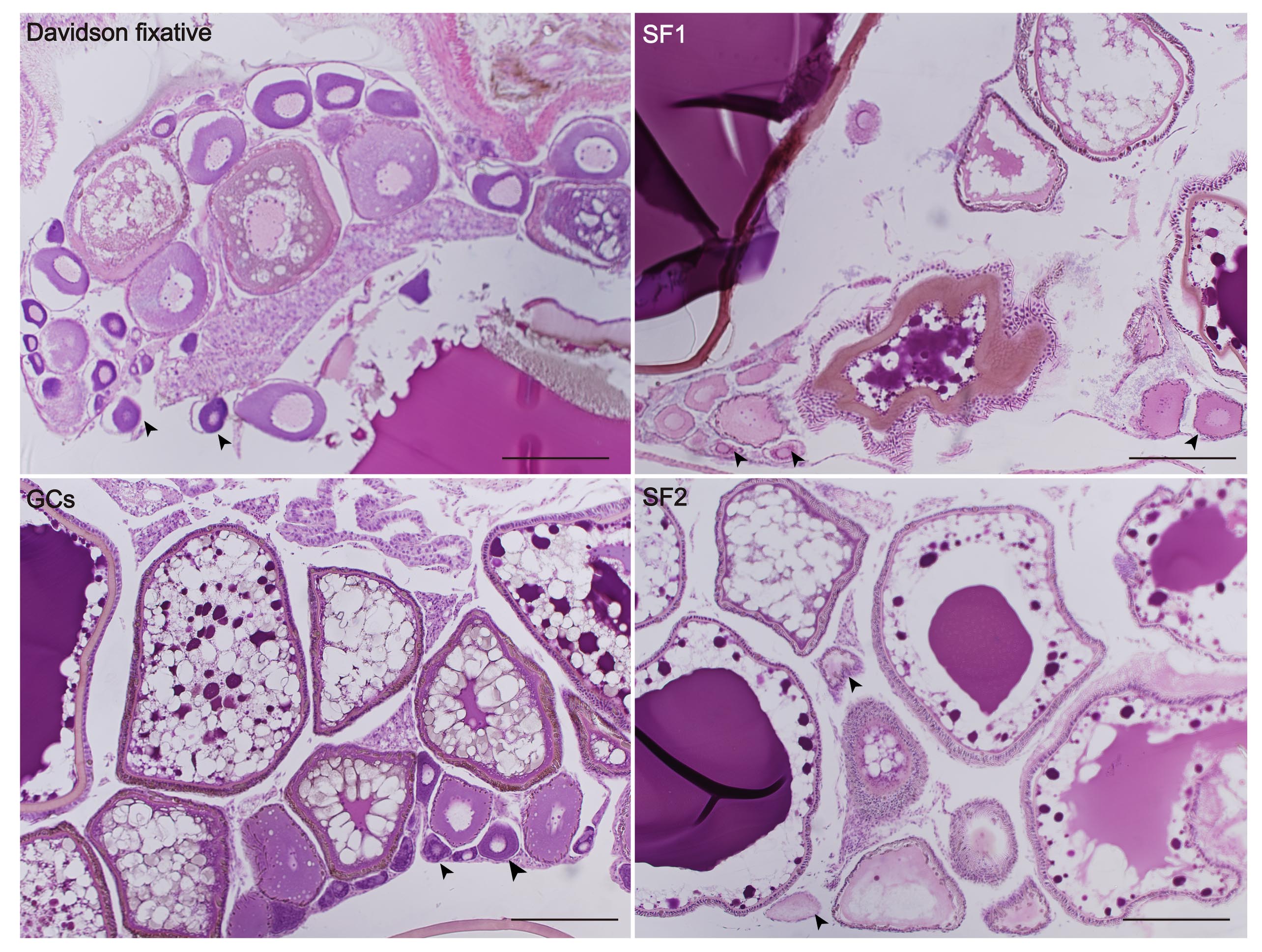

Supplement: S3 Fig — Two female fish in the GC group and three in the SF group were fixed with PFA. Fig 1 presents one GC samples and one SF sample, with the rest being shown in this figure. Arrowheads showed the previtellogenic stage oocytes. Scale bars = 50 μm. (JPG) [file pone.0138799.s003.jpg]

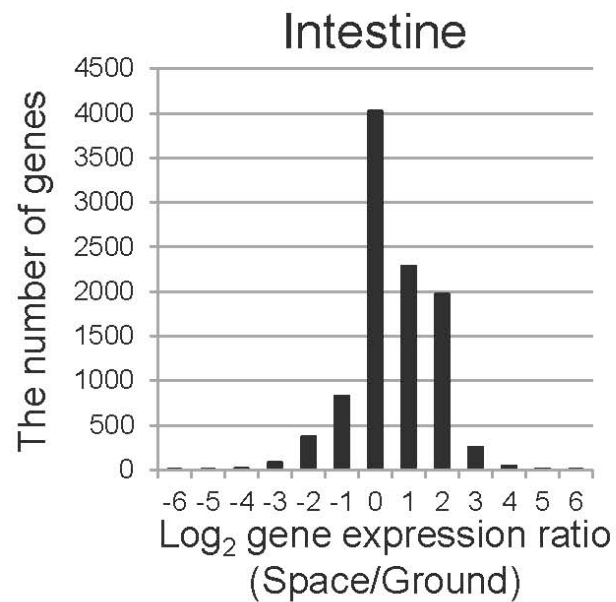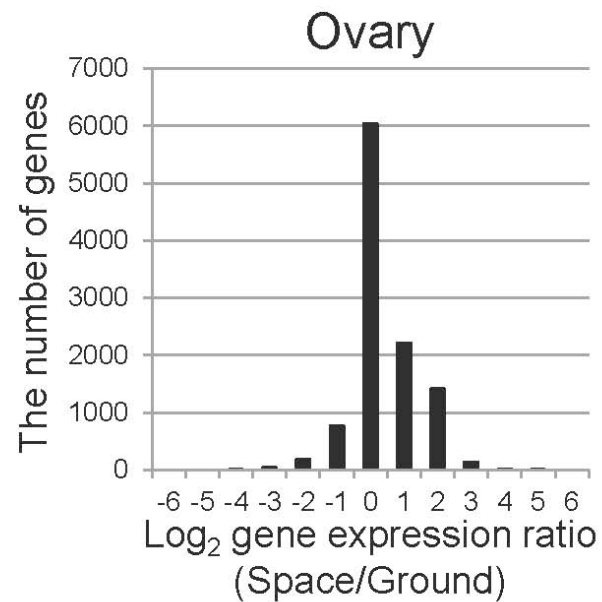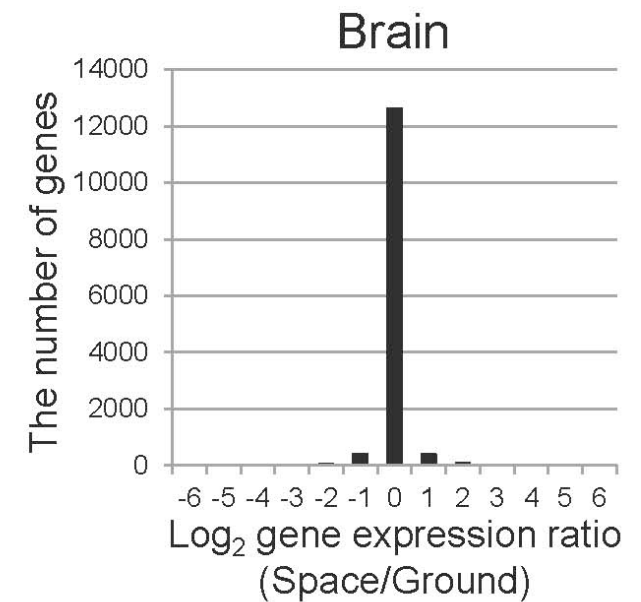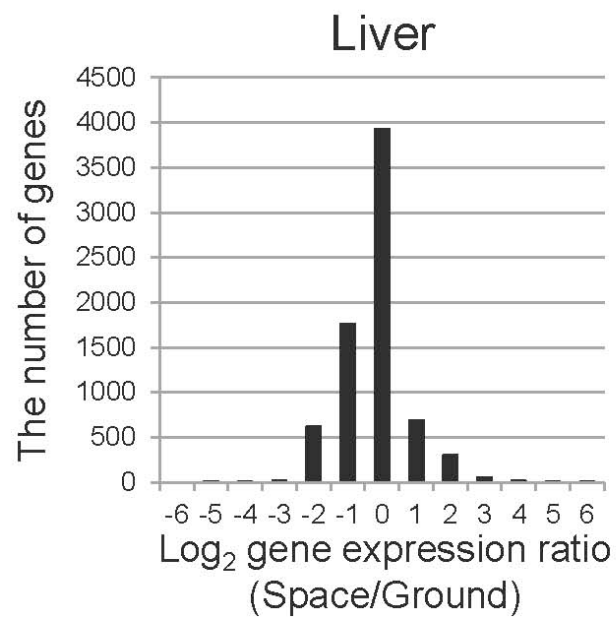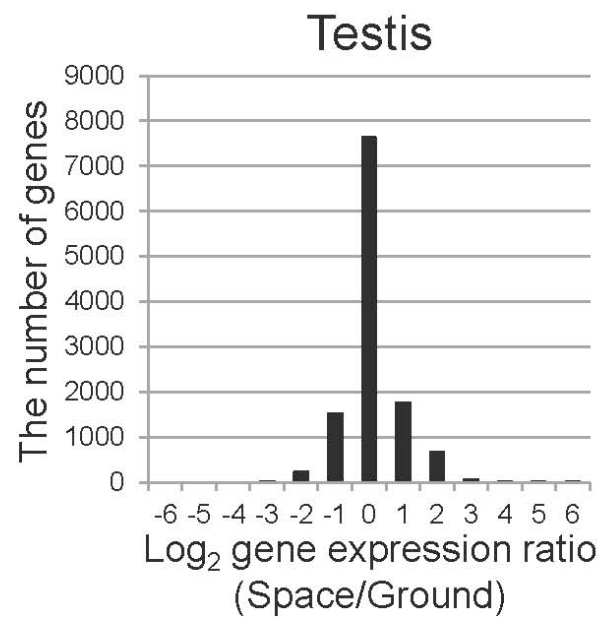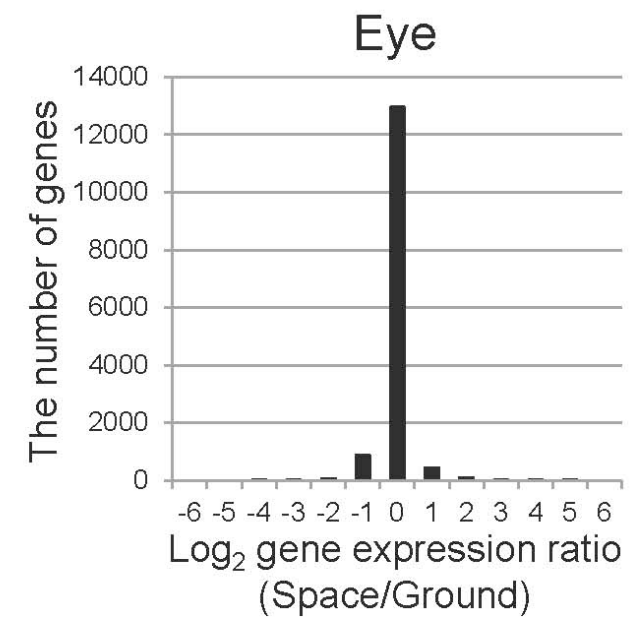

Supplement: S4 Fig — Gene expression analyses were conducted for the caudal part of the intestine, and the liver, ovary, testis, brain, and eyes. (PDF) [file pone.0138799.s004.pdf]
